# The Tolerance Differences of Two Industrial Hemp Varieties Under Lead (Pb) Stress

**DOI:** 10.3390/toxics13020090

**Published:** 2025-01-24

**Authors:** Yanping Xu, Anuwat Kumpeangkeaw, Xia An, Xuan Chen, Yuan Zhang, Pin Lv, Qingying Zhang, Rong Guo, Qingqing Ji, Ming Yang

**Affiliations:** 1 Industrial Crops Research Institute, Yunnan Academy of Agricultural Sciences, Kunming 650205, China; chenxuan9239@163.com (X.C.); 136151701@163.com (Y.Z.); lvpinhemp@163.com (P.L.); hempzhangqingying@126.com (Q.Z.); grmm0207@126.com (R.G.); 2 Yunnan Key Laboratory of Genetic Improvement of Herbal Oil Crops, Kunming 650205, China; 3 Department of Agriculture, Ministry of Agriculture and Cooperatives, Bangkok 10900, Thailand; flaaaay66@gmail.com; 4 Zhejiang Xiaoshan Institute of Cotton & Bast Fiber Crops Research, Zhejiang Institute of Landscape Plants and Flowers, Zhejiang Academy of Agricultural Science, Hangzhou 311251, China; anxia@zaas.ac.cn (X.A.); jiqingqing@stu.ynu.edu.cn (Q.J.); 5 School of Agriculture, Yunnan University, Kunming 650500, China

**Keywords:** industrial hemp, lead (Pb), tolerance differences, lead accumulation

## Abstract

Industrial hemp is a crop with a high tolerance and accumulation of lead (Pb). Improving the Pb tolerance and accumulation capacity of industrial hemp is of great scientific and practical importance. This study utilized a pot with soil contaminated with Pb to investigate the differences in Pb tolerance between two industrial hemp varieties, Yunma1 (YM) and Shaanxi Industrial Hemp (SM), under Pb stress. The results indicated that Pb mainly accumulates in the roots of YM and SM (70–80%), with YM having a higher Pb accumulation than SM. It is worth nothing that under high Pb concentration conditions (5000 mg/kg), the Pb accumulation capacity of YM is twice that of SM. Accumulation characteristics of Pb in different plant tissues followed the pattern: roots > stems > leaves > fibers > seeds. In YM, approximately 70% of the absorbed Pb was fixed in the roots and 30% was transported to the above-ground parts. In contrast, SM transported more than 50% of absorbed Pb by roots to the above-ground areas, causing some degree of damage to stems and leaves. Even when Pb concentrations exceed 4000 mg/kg, YM exhibits strong tolerance (tolerance index greater than 90%), with normal growth and no signs of toxicity. However, SM showed a tolerance level of < 50% at high Pb concentrations, with significant heavy metal toxicity symptoms in the above-ground areas. These results provide important information for the remediation of Pb contaminated soils in mining areas.

## 1. Introduction

In many terrestrial and aquatic ecosystems, the issue of heavy metal stress has become a major global concern. Due to the heavy metal accumulation, extensive industrialization in the recent past has been shown to have negative effects on agricultural productivity and soil quality [1]. Heavy metal, like lead (Pb), mercury (Hg), cadmium (Cd), arsenic (As), chromium (Cr), and nickel (Ni), have harmful effects on agricultural ecosystems. Due to their non-biodegradable features, they become a threat factor for human health and soil ecosystems [2,3]. Lead, as a heavy metal element typically toxic to plants, enters animal and plant systems through atmospheric deposition and soil absorption [3,4,5,6]. In plants, lead absorption disrupts critical physiological processes such as seed germination, root growth, and chlorophyll synthesis [7,8]. For instance, Pb was absorbed by the roots of hemp, and accumulated in the grains of the rice [9,10]. Pb lowers the photosynthetic rate of plants by damaging chloroplast ultrastructure, reducing chlorophyll production, blocking electron transport, and suppressing Calvin cycle enzyme activity [11]. Pb stress interferes with the plant’s normal physiological processes. It suppresses enzyme activity, water balance, mineral nutrition, photosynthesis, and may lead to cell death [12]. However, the extent of lead accumulation, tolerance, and inhibition varies between plant species and even among varieties of the same species.

*Cannabis sativa* L. is an annual herbaceous plant of the *Cannabaceae* family, traditionally cultivated as an economic crop [13]. It is widely used in industrial, agricultural, and pharmaceutical sectors. Industrial hemp, a genetically improved variant with tetrahydrocannabinol (THC) levels below 0.3%, is distinguished by its short growth cycle, high biomass production, robust root system, adaptability, and ability to accumulate various heavy metals [9,14]. These characteristics not only confer high economic value but also highlight its significant potential for remediating heavy metal-contaminated soils [15].

The taproot of industrial hemp absorbs a large amount of non-nutrient elements from the soil during growth [16,17,18]. For example, studies by Golia et al. and Xu et al. demonstrated the strong Pb accumulation capacity of industrial hemp, making it uniquely advantageous for remediating heavy metal-contaminated soils [19,20]. Differences in Pb accumulation traits and tolerance mechanisms among various industrial hemp cultivars have not, however, been thoroughly studied.

Industrial hemp is widely recognized as an excellent potential crop for the remediation of heavy metal contaminated soils. However, previous studies have focused primarily on on-site remediation of field contamination. For example, Luyckx et al. (2022) replaced industrial hemp with bioenergy in lead-contaminated farmland in northern France by growing crops and spraying silicon on the pages to increase the heavy metal content of the crops and to improve and maintain the mechanical properties of the fibers [21]. Flajšman et al. (2023) investigated the uptake of heavy metals, such as Pb, by two industrial hemp varieties in areas with different levels of contamination, and found that the plant uptake of lead was highest in heavily contaminated soils [22]. The soils in these studies contained multiple heavy metals at the same time, and the natural environment was subject to a variety of uncontrollable factors that affected the experimental results. In view of this, this study focuses on two industrial hemp varieties with differing Pb tolerance levels. These two varieties originate from southern (YM) and northern (SM) part of China. This study examines the impact of Pb on the growth of these two species using pot experiments with artificially administered Pb to resemble natural environmental Pb stress. The study also looks at how different plant components absorb and accumulate Pb. Investigating the mechanisms of industrial hemp’s tolerance to Pb stress and providing theoretical insights into its safe application in Pb-affected farmlands, soil restoration, and environmental enhancement are the objectives.

## 2. Materials and Methods

### 2.1. Experimental Materials

Two industrial hemp materials were used to study differences in Pb tolerance: Yunma 1 (YM), a Pb-tolerant, high-biomass, late-maturing variety bred by the Economic Crops Research Institute of the Yunnan Academy of Agricultural Sciences, and Shaanxi Industrial Hemp ym478 (SM), a Pb-sensitive, low-biomass, early-maturing variety. The test soil is red loam, derived from the cultivated layer (0–20 cm) at the experimental base of the Industrial Crops Research Institute of Yunnan Academy of Agricultural Sciences, Panlong District, Kunming City, Yunnan Province, China. The physicochemical properties of soil are presented by Inductively Coupled Plasma Mass Spectrometry (ICAP RQ, Thermo Fisher Scientific, Waltham, MA, USA) (Table 1).

### 2.2. Experimental Design

The pot experiment was carried out in the greenhouse of the Industrial Crops Research Institute of Yunnan Academy of Agricultural Sciences. Analytical grade Pb (NO_3_)_2_ was used as the treatment agent. Seven Pb^2^⁺ concentration levels were established, corresponding to soil Pb concentrations of 0 (65), 500 (647), 1000 (1468), 2000 (2497), 3000 (3275), 4000 (4861), and 5000 (5676) mg/kg. There were three “replicated“ treatments. Each copy was a pot with a diameter of 50 cm, a height of 30 cm, and contained 21 kg of soil. After the addition of Pb, the soil was equilibrated for 15 days by 3 saturation cycles with distilled water and dry air. pot was given 2.35 g Potassium, 6.18 g Superphosphate, and 3.82 g Urea Sulfate as the base fertilizer. 20 seeds were sowed in each pot and 5 seedlings were kept after germination. After sowing, they were watered once a week, using 5 L of tap water per pot. Industrial hemp is grown at 18–25° with 13 h of sunlight in the day and 75% relative humidity. When the industrial hemp grows to the 3–4 pairs of true leaves stage, each pot is given 10 g of urea as a follow-up fertilizer. YM and SM plants reached seed maturity at 180 and 150 days, respectively. Plants were hand-harvested whole, dried naturally. Five plants per pot were sampled for traits, biomass, and metal content. All measurements were repeated three times. The roots of harvested plant samples were washed three times with tap water, then three to four times with distilled water, and finally vacuumed with absorbent paper. Each sample was re-watered to avoid cross-contamination of roots, stems, and leaves with soil.

### 2.3. Experimental Methods

#### 2.3.1. Measurement of Agronomic Traits and Biomass of Two Industrial Hemp Varieties

A vernier caliper was used to measure stem diameter at harvest, and a measuring tape was used to measure plant height and root length. The biomass of the various plant parts was ascertained by measuring the dry weights of roots, stems, leaves, seeds, and fibers after the harvested plants were allowed to air dry naturally.

#### 2.3.2. Determination of Pb Content in Samples

The dried plant samples were quenched at 105 °C for 0.5 h and dried in an oven at 70 °C to a constant weight, ground in an agate mortar with liquid nitrogen, and passed through a 0.42 mm sieve. They were weighed at approximately 0.2–0.5 g (accurate to 0.0001 g) with an electronic balance, then 5–10 mL of 68% nitric acid was added, they were covered and left for 1 h or overnight, according to the microwave digestion instrument (GB 5009.268-2016 National Standard for Food Safety Determination of Multi-Elements in Food Table B.1) operation. After cooling, ultrapure water of a volume up to 25 mL was used, then a blank test was performed.

ICP-MS (ICAP RQ, Thermo Fisher Scientific, Waltham, MA, USA) inductively coupled plasma mass spectrometry (GB-5009.268-2016 National Standard for Food Safety Determination of Multiple Elements in Foods First Method) was used to determine the lead content.

#### 2.3.3. Calculation of Accumulation and Tolerance Capacity in Industrial Hemp

The bioconcentration factor (BCF), which measures how well plants absorb heavy metals, was used to evaluate the plants’ ability to accumulate heavy metals. It was found that the plant’s capacity to absorb heavy metals was directly proportional to their bioconcentration factor (BCF) values [23,24]. The effectiveness of moving heavy metals from the roots to the above-ground organs is gauged by the translocation factor (TF), whose value shows how the metals are distributed and accumulate inside the plant.

The tolerance index (TI) is a key indicator of a plant’s resistance to heavy metals, directly reflecting plant physiological adaptation to heavy metal stress. According to Lux et al., plants can be classified into three categories based on their TI values: highly tolerant (TI > 60%), moderately tolerant (35% ≤ TI ≤ 60%), and sensitive (TI < 35%) [25]. It is further noted that when plant biomass is reduced to 50% of the control group (i.e., TI = 50%), this can be considered the toxicity threshold for heavy metals, marking the critical point at which a plant transitions from tolerance to sensitivity [26]. Bioconcentration factor (BCF) = heavy metal content in a specific plant organ/heavy metal content in rhizosphere soil, translocation factor (TF) = heavy metal content in a specific plant organ/heavy metal content in the transport organ of the plant, and tolerance index (TI) = (Biomass of industrial hemp under Pb treatment/Biomass of control group industrial hemp) × 100%.

### 2.4. Data Statistical Analysis

SPSS 20.0 was used to analyze the experimental data, and GraphPad Prism 8 was used to visualize the results.

## 3. Results

### 3.1. The Effect of Pb Stress on Agronomic Traits of Industrial Hemp

The normal growth of plants can be indicated by their agronomic characteristics. In Pb-contaminated soils, the most directly impacted part is the root system, and root length, to some extent, reflects the development, growth, and metabolic status of the root system. After Pb is absorbed by roots, it will affect the plant’s height and stem diameter; therefore, plant height, stem diameter, and root length are key indicators of the growth vigor of industrial hemp.

The effects of Pb stress on the growth of the two industrial hemp varieties are shown in Table 2. With an increase in Pb concentration, the plant height, stem diameter, and root length of both varieties initially increased and then decreased. Low concentrations of Pb exhibited a growth-promoting effect, whereas higher concentrations had an inhibitory effect on plant height and stem diameter. For YM, plants demonstrated strong growth within a Pb concentration range of 0–4000 mg/kg. However, when the concentration increased to 5000 mg/kg, plant growth was moderately inhibited. Despite this, the overall growth process continued to be normal. Compared to the control group, plant height decreased by roughly 15–17 cm, with notable changes seen as relative to other treatments. In addition, both stem diameter and root length decreased. At the seed maturity stage, no symptoms of heavy metal toxicity or plant death were observed, indicating that YM exhibited strong self-repair capacity. SM, the plant height, stem diameter, and root length followed a similar trend of initially increasing and then decreasing with increasing Pb concentration, peaking at a Pb concentration of 1000 mg/kg. When the Pb concentration reached 4000 mg/kg, these growth parameters were significantly inhibited, showing notable differences compared to the control group (CK). At a Pb dose of 5000 mg/kg, the height, stem diameter, and root length of SM decreased by 12.16%, 13.99%, and 13.01%, respectively. Compared with SM, YM has superior resistance to Pb stress.

### 3.2. Effects of Different Pb Concentrations on the Biomass of Industrial Hemp Organs

**Figure 1:**
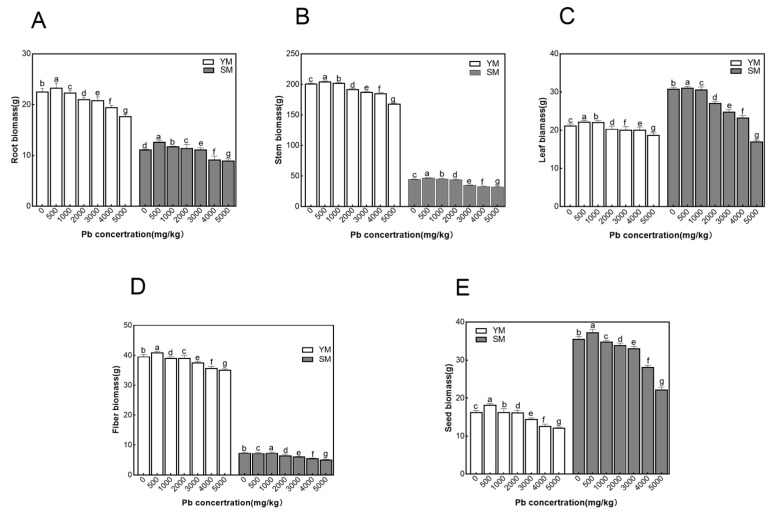
Biomass of different cultivars under Pb stress. (**A**): Comparison of root biomass; (**B**): Comparison of stem biomass; (**C**): Comparison of leaf biomass; (**D**): Comparison of fiber biomass; (**E**): Comparison of seed biomass. Data in the figure are mean ± standard, different lowercase letters indicate significant differences between different concentrations (*p* < 0.05).

The inhibitory effects of Pb stress on the biomass of different industrial hemp organs varied with Pb concentrations (Figure 1). At a low Pb concentration (500 mg/kg), the biomass of YM and SM organs was slightly higher than that of the control group, indicating that low Pb concentrations may have a stimulating impact on industrial hemp development of industrial hemp. With increase in Pb concentration, the biomass of industrial hemp organs demonstrated a decreasing tendency, which was significantly different from the control group.

For root biomass (Figure 1A) in the Pb concentration range of 500–1000 mg/kg, there was no significant difference between the two varieties and the control group. As the Pb content increased, the root biomass of both types gradually decreased. At Pb concentrations of 2000–4000 mg/kg for YM and 2000–3000 mg/kg for SM, significant differences (*p* < 0.05) were observed in root biomass compared to the control. However, the downward trend was not dramatic. However, under Pb stress at 5000 mg/kg for YM and 4000 mg/kg for SM, root growth was significantly inhibited. At these concentrations, root biomass decreased by 21.56% for YM and 17.47% for SM compared to control. The root biomass of YM was more than twice that of SM.

For stem and leaf biomass (Figure 1B,C), as the Pb concentration of the stems increased from 500 to 4000 mg/kg, YM biomass showed a statistically significant downward trend (*p* < 0.05), although the reduction was not significant. At a concentration of 5000 mg/kg, stem biomass was significantly impacted, showing a reduction of 16.45% compared to the control group. In SM, stem biomass showed no significant variation from control at Pb doses ranging from 500 to 2000 mg/kg. At concentrations above 3000 mg/kg, significant differences (*p* < 0.05) were observed compared to control. For leaves, low Pb concentrations (500–1000 mg/kg) did not significantly affect biomass compared to the control. At doses above 2000 mg/kg, Pb significantly impeded leaf growth, affecting biomass accumulation. At 5000 mg/kg, leaf biomass decreased by 44.67% relative to control, presumably due to Pb-induced cellular damage, which compromised photosynthetic efficiency. For fiber and seed biomass (Figure 1D,E), Pb stress had different effects on the accumulation of fiber and seed dry matter in YM and SM, especially at high Pb concentrations (5000 mg/kg). Compared with the control, the biomass of YM fibers and seeds decreased by 11.3% and 25.0%, respectively, but SM showed reductions of 31.1% and 31.7%, indicating a substantial inhibition of SM fibers and seeds. Compared to SM, YM showed greater resilience under elevated Pb stress. This was evident from its reduced biomass reduction across multiple organs, especially roots, fibers, and seeds.

### 3.3. Accumulation Characteristics of Pb in Two Industrial Hemp Varieties

**Figure 2:**
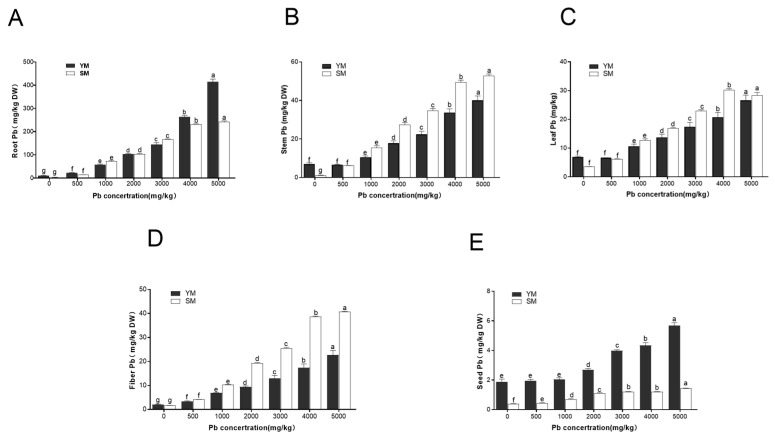
Pb content in different organs of two industrial hemp varieties under Pb stress (mg/kg). (**A**): Comparison of Pb content in roots; (**B**): Comparison of Pb content in stems; (**C**): Comparison of Pb content in leaves; (**D**): Comparison of Pb content in seeds; (**E**): Comparison of Pb content in fibers. Different lowercase letters represent significant differences in different concentrations (*p* < 0.05).

As the Pb treatment concentration increased, the Pb content in all plant organs also increased, although the rate of increase varied significantly, with roots showing a much greater increase than other organs (Figure 2). Pb accumulation in different organs followed the pattern: roots > stems > leaves ≈ fibers > seeds. Lead was mainly contained in roots, followed by stems, leaves, and fibers, with the least amount found in seeds. However, the deposition of Pb varied significantly between the two materials. In YM, the concentration of Pb in roots was 1–20 times greater than in stems, leaves, and fibers, and 5–70 times higher than in seeds. In SM, when Pb concentration increased, Pb accumulation in roots progressively migrated to aerial organs, leading to elevated Pb levels in stems, leaves, and fibers. In SM, the Pb concentration in roots was only 0.17–0.43 times than that in stems, 1–10 times than that in leaves and fibers, and 8–170 times than that in seeds. In both materials, the lead level in the seeds was minimal; even at a lead concentration of 5000 mg/kg, the lead content in the seeds did not exceed 10 mg/kg. At soil Pb values below 2000 mg/kg, no significant variation in root Pb content was observed between YM and SM. However, when the concentration exceeded 3000 mg/kg, the Pb level in YM roots was significantly higher than in SM roots. At a dose of 5000 mg/kg, the lead content in YM roots was approximately twice that of SM roots, measuring 414.33 mg/kg and 242.70 mg/kg, respectively (Figure 2A). Figure 2B–D illustrate that at low Pb concentrations (0–500 mg/kg), Pb levels in stems, leaves, and fibers of both materials showed no significant differences. At values of 1000 mg/kg and above, the Pb content in YM was lower than that in SM. At 5000 mg/kg, Pb levels in the SM stems, leaves, and fibers were 1.34, 1.06, and 1.96 times higher than YM, respectively. In seeds (Figure 2E), the Pb concentration in both YM and SM was minimal (<10 mg/kg), although the Pb concentration in YM was significantly higher than in SM.

### 3.4. Effects of Pb Stress on the Accumulation Capacity of Different Parts of the Two Industrial Hemp Varieties

**Figure 3:**
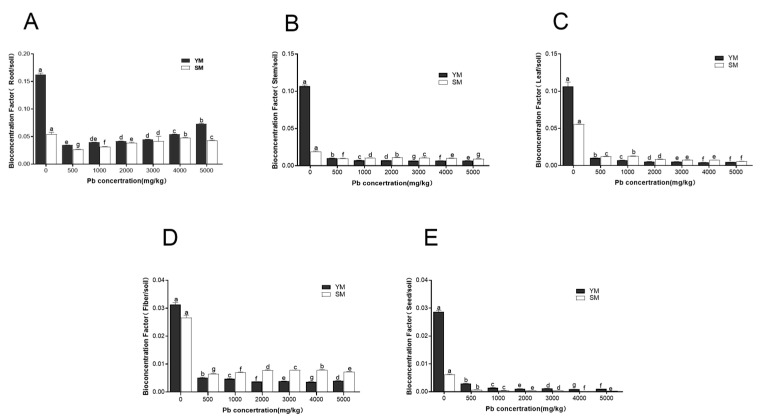
Bioconcentration factor of Pb from different organs in two industrial hemp varieties. (**A**): Root bioconcentration factor; (**B**): Stem bioconcentration factor; (**C**): Leaf bioconcentration factor; (**D**): Seed bioconcentration factor; (**E**): Fiber bioconcentration factor. Different lowercase letters represent significant differences in different concentrations (*p* < 0.05).

As shown in Figure 3, significant differences were observed in bioconcentration factors (BCFs) for roots, stems, leaves, fibers, and seeds of the two industrial hemp varieties. BCFs in roots were consistently higher than those in other organs. For both YM and SM, root BCFs decreased gradually as Pb concentration increased, with significant differences observed between treatments (*p* < 0.05). YM root BCF was higher than SM, especially at Pb concentrations of 0 mg/kg and 5000 mg/kg, where YM root BCF was 2.99 and 1.71 times that of SM, respectively.

Figure 3B–D illustrate that BCFs in stems, leaves, and fibers for both YM and SM decreased to different extents as Pb concentration increased, with statistically significant differences between treatments (*p* < 0.05). In contrast to roots, YM BCFs in stems, leaves, and fibers were inferior to SM, except for the control group. Figure 3E shows that bioaccumulation factors (BCFs) for seeds in both YM and SM were low (BCF < 0.03). However, YM seed BCF was significantly higher than SM seed. While Pb mainly accumulated in the roots of both types, their bioconcentration capacities significantly. YM exhibited the ability to absorb significant amounts of Pb and withstand elevated Pb concentrations (5000 mg/kg). The bioconcentration capacity of SM decreased dramatically at a Pb concentration of 5000 mg/kg. YM showed significantly greater bioconcentration capacity in roots and seeds compared to SM, but SM exhibited superior BCFs in stems, leaves, and fibers compared to YM.

### 3.5. Effects of Pb Stress on the Translocation Ability of Different Parts of Two Industrial Hemp Varieties

**Figure 4:**
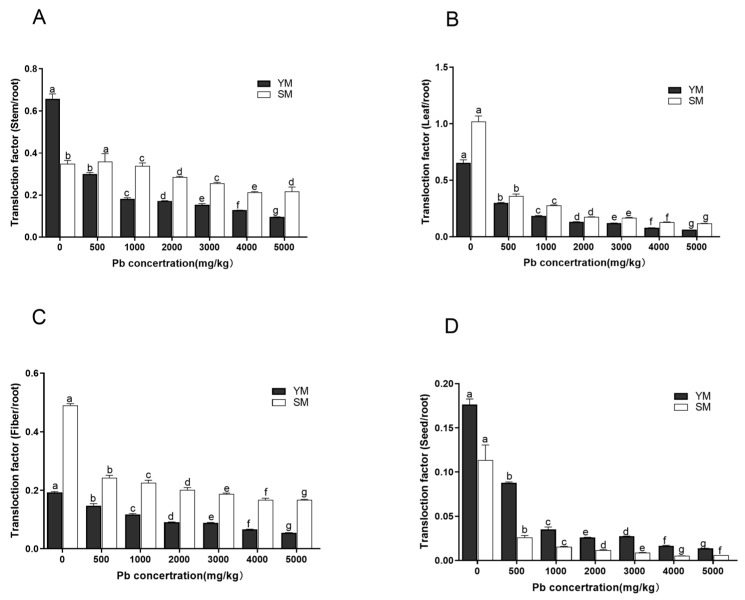
Translational factors of Pb form different organs in two industrial hemp varieties. (**A**): Stem translocation factor; (**B**): Leaf translocation factor; (**C**): Fiber translocation factor; (**D**): Seed translocation factor. Different lowercase letters represent significant differences in different concentrations (*p* < 0.05).

Figure 4 demonstrates that Pb transfer coefficients (TFs) for stems, leaves, fibers, and seeds in both types of industrial hemp decreased progressively with elevated Pb concentrations, with significant variations seen between treatments (*p* < 0.05). Transcription factors for SM stems (excluding the control group) and leaves were elevated compared to YM (Figure 4A,B). This suggests that SM has a superior ability to translocate Pb from roots to aerial stems and leaves compared to YM. This may explain why SM leaves exhibited chlorosis and biomass loss at a Pb concentration of 4000 mg/kg, indicative of toxic symptoms. TFs for both YM and SM fibers decreased gradually as Pb concentration increased, with significant differences between treatments (*p* < 0.05). Although TFs for both varieties were generally low (TF < 0.6), TF for fibers were significantly lower than SM fibers (Figure 4C). This suggests that a large amount of Pb transferred from the roots to stems in SM accumulated in bast fibers, causing severe damage to stem tissue.

Correspondingly, seed transfer factors for both YM and SM decreased as Pb levels increased with significant differences between treatments (*p* < 0.05). Transcription factors (TFs) for seeds in both types were low (TF < 0.2), although YM exhibited significantly stronger TFs in seeds than SM (Figure 4D). In summary, SM exhibited a significantly superior ability to translocate Pb to stem, leaf, and fiber relative to YM, which likely resulted in increased damage observed in SM under elevated Pb stress. In contrast, YM had a superior ability to transfer Pb to seed relative to SM.

### 3.6. Comparison of Pb Tolerance Indices in Two Industrial Hemp Varieties

**Figure 5:**
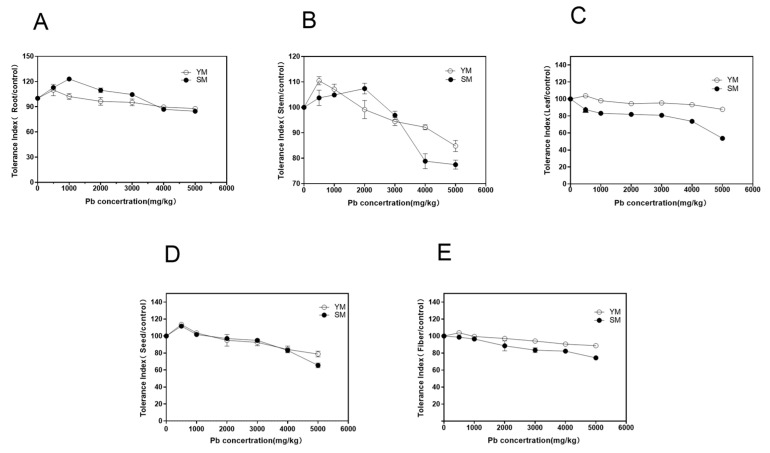
Tolerance index in different organs of two industrial hemp varieties under Pb stress. (**A**): Comparison of root tolerance index; (**B**): Comparison of stem tolerance index; (**C**): Comparison of leaf tolerance index; (**D**): Comparison of seed tolerance index; (**E**): Comparison of fiber tolerance index. The error line in the figure represents the mean ± standard.

As the concentration of Pb increased, the root tolerance index (TI) initially increased and then decreased, with variations between the two varieties (Figure 5A). At a low Pb concentration (500 mg/kg), there was little difference in YM and SM root TIs, and both were higher than the control. Between 1000 and 3000 mg/kg, the TI for YM roots was lower than for SM. At concentrations of 4000–5000 mg/kg, both varieties exhibited root TIs above 60%, with YM showing slightly higher TIs than SM. This indicates that the roots of both varieties have a high tolerance to heavy metal Pb.

For stems, the TI for YM was higher than the control at a low Pb concentration (500 mg/kg). As the concentration of Pb increased, TI gradually decreased, although the decrease was small, and TI remained above 60% across all concentrations (Figure 5B). In SM, stem TI increased as Pb concentration increased to 2000 mg/kg but began to decrease at concentrations above 2000 mg/kg. At 4000–5000 mg/kg, TI decreased to its lowest level and below YM, indicating that SM stems have a lower tolerance to Pb in high concentration environments compared to YM.

For leaves, YM showed a slow increase in TI as Pb concentration rose to 3000 mg/kg. At doses of 4000–5000 mg/kg, TI showed only a marginal decline, maintaining above 90% even at 5000 mg/kg, indicating significant Pb tolerance in YM leaves (Figure 5C). TI for SM leaves was below 50% at 5000 mg/kg. Wang et al. [26] indicates that a toxicity index of 50% serves as the threshold for heavy metal toxicity in plants. At a concentration of 5000 mg/kg, SM leaves showed a 50% decrease in biomass, indicating that the soil lead toxicity threshold for SM leaves is 5000 mg/kg. For seeds and fibers, YM showed higher TIs than control at a low Pb dose (500 mg/kg). As Pb concentration increased, TIs gradually decreased but remained above 60% at all concentrations (Figure 5D,E). However, the tolerance index for SM seeds at 5000 mg/kg was lower than that for YM seeds, suggesting that YM seeds exhibit more tolerance to Pb than SM seeds.

Overall, YM showed significant tolerance to Pb, with translocation indices for roots, stems, leaves, seeds, and fibers consistently exceeded 60% at all concentrations. In SM, TIs surpassed 60% at low Pb concentrations but decreased significantly in high Pb conditions. Notably, SM leaves showed a 50% TI at 5000 mg/kg, confirming the soil Pb toxicity threshold for SM leaves to be 5000 mg/kg. YM consistently shows superior tolerance to Pb in all plant organs relative to SM.

## 4. Discussion

### 4.1. Differences in Pb Tolerance Response Between Two Industrial Hemp Materials

Depending on the plant species and the specific organs involved, different plants have different capacities for absorbing and accumulating heavy metals [27,28,29,30,31,32,33,34,35]. For example, plants such as ramie, pepperwood, paper mulberry, and black nightshade mainly fix Pb in their roots, limiting their transport to parts above ground. This reduces damage to photosynthetic tissues and respiratory systems in above-ground organs, thereby enhancing Pb tolerance [36,37].

Pb is mostly accumulated in the roots of SM and YM cultivars in our investigation. Nevertheless, significant variations in Pb accumulation were noted between the two types. The Pb concentration in YM roots was twice that of SM roots under heavy Pb stress (>4000 mg/kg). YM showed that it could withstand elevated Pb concentrations and continue to grow normally under such circumstances. On the other hand, SM showed severe signs of heavy metal toxicity, including leaf chlorosis, poor seed development, hollow seeds, and premature aging, at comparable high Pb stress levels. The differences can be explained by the fact that YM retains a significant amount of lead (about 70%) in its roots, while only a small part (about 20%) is transferred to organs above ground. Pb-tolerant industrial hemp uses this retention mechanism as one of its methods to reduce heavy metal stress [38,39,40]. In contrast, SM caused high Pb concentrations in stems, leaves, and fibers by moving a large amount of Pb absorbed by the roots (>50%) to above-ground areas. SM may have a reduced tolerance to Pb due to the damage to terrestrial organs caused by this excessive accumulation, resulting in signs of Pb contamination. Although the bioconcentration factors (BCFs) and translocation factors (TFs) of both varieties did not meet the criteria for hyperaccumulator plants as proposed by Baker and Brooks [41] (BCF and TF > 1, Pb concentration in above-ground areas >1000 mg/kg, and no impact on normal plant growth under heavy metal stress), industrial hemp exhibited high biomass. In addition, YM showed no apparent signs of Pb toxicity under high Pb stress and showed higher tolerance and accumulation capacity than SM. Depending on local conditions, industrial hemp materials can be used selectively for the restoration of Pb contaminated soils in mining sites because of the different Pb accumulation and translocation capacities of both types.

### 4.2. Differences in Lead Tolerance Between Two Industrial Hemp Varieties

Plants’ ability to acquire metals is only one aspect of their tolerance to heavy metals; another is their ability to continue growing normally in the face of heavy metal stress. As a key determinant of typical plant growth, biomass fully captures a plant’s ability to store and use resources and energy [42,43]. According to Kandziora-Ciupa et al. [44], biomass allocation is a plant’s method of balancing reproduction and survival while adjusting to its external environment. Increased biomass leads to a higher accumulation of heavy metals at constant concentrations. However, plant growth and development are impeded when heavy metals accumulate above a certain threshold. Arthur et al. [45] classified crops into high, medium, and low accumulators based on heavy metal accumulation levels. In this study, significant differences were observed between YM and SM in morphology, biomass, and tolerance to Pb stress.

1. Morphological differences: Plant height, stem diameter, and root length were somewhat suppressed in YM at Pb concentrations of 4000–5000 mg/kg, but no contamination symptoms were observed. In contrast, SM exhibited severe toxicity symptoms, including leaf chlorosis, poor seed development, hollow seeds, and premature aging. This phenomenon of “low promotion, high inhibition” under Pb stress is consistent with findings in other plants, such as Sedum alfredii, where low Pb concentrations promote growth while high concentrations inhibit growth [46,47].
2. Biomass differences: YM biomass was more than 2.5 times larger than SM biomass. When soil Pb concentration was less than 1000 mg/kg, industrial hemp growth was promoted, with higher root and leaf biomass compared to the control group. However, biomass accumulation and plant development were inhibited at elevated Pb concentrations. Both YM and SM showed a considerable inhibition of root growth at Pb values of 5000 mg/kg and 4000 mg/kg. At doses as low as 3000 mg/kg, the biomass of SM differed significantly from control (*p* < 0.05), and at 5000 mg/kg, it decreased by more than 50%, suggesting a severe suppression of plant growth.
3. Tolerance differences: The tolerance indices (TIs) for roots and stems in both YM and SM exceeded 60%. However, at high Pb concentrations (>4000 mg/kg), TI for YM was significantly higher than for SM. In leaf tissue, YM exhibited higher TIs than SM, with TI exceeding 90% even at 5000 mg/kg. However, at Pb concentrations above 5000 mg/kg, SM showed a TI < 50%, suggesting that SM leaves have a Pb toxicity threshold of 5000 mg/kg. Industrial hemp is classified as a medium accumulator with high tolerance based on the accumulation characteristics seen in YM and SM in this study as well as the criteria established by Arthur [45] for dividing crops into high, medium, and low accumulators of heavy metals. In addition, YM showed greater Pb tolerance than SM.

## 5. Conclusions

Industrial hemp, as a fiber crop not used for consumption, ensures that heavy metals do not enter the food chain, making it an excellent candidate for remediating heavy metal contaminated soils. Regarding Pb accumulation in tissues, Pb mainly accumulates in roots of both YM and SM varieties, with YM showing a higher accumulation capacity than SM. Only 30% of the Pb absorbed by YM was transferred to the above-ground portions; the remaining 70% was sequestered in its roots. On the other hand, under high Pb conditions, SM caused toxicity symptoms by translocating 50% of the Pb that had accumulated in the roots to its stems and leaves. Industrial hemp is classified as a medium accumulator with exceptional Pb tolerance by combining phenotypic, biomass, and Pb accumulation and translocation assessments. When it comes to Pb tolerance, YM is more resilient than SM.

## Figures and Tables

**Table 1:** Physical and chemical properties of the tested soil.

pH	Organic Matter (g/kg)	Total Nitrogen (N, %)	Total Phosphorus (P, %)	Total Potassium (K, %)	Hydrolyzable Nitrogen	Available Phosphorus	Available Potassium	Total Lead
					(N, mg/kg)	(P, mg/kg)	(K, mg/kg)	(Pb, mg/kg)
6.69	97.5	0.392	0.213	0.176	263	27.6	642	65.03

**Table 2:** Agronomic traits of different hemp cultivars under Pb stress.

Varieties	Pb (mg/kg)	Plant Height (cm)	Stem Diameter (mm)	Root Length (cm)
YM	0	310.3 ± 2.1 b	10.2 ± 0.2 b	28.3 ± 0.5 a
	500	328.3 ± 5.9 a	12.5 ± 0.4 a	28.7 ± 0.6 a
	1000	308.1 ± 4.2 bc	10.0 ± 0.1 bcd	27.3 ± 0.3 b
	2000	303.2 ± 2.0 cd	10.3 ± 0.7 bc	26.6 ± 0.3 b
	3000	302.0 ± 3.0 d	9.7 ± 0.3 cd	25.8 ± 0.2 c
	4000	295.7 ± 4.0 e	9.5 ± 0.5 d	25.6 ± 0.5 c
	5000	293.0 ± 3.0 e	9.4 ± 0.5 d	22.9 ± 0.3 d
SM	0	148.7 ± 6.1 ab	6.8 ± 0.3 b	14.0 ± 0.5 ab
	500	149.7 ± 4.8 ab	7.0 ± 0.1 b	14.3 ± 0.5 ab
	1000	155.5 ± 5.3 a	7.6 ± 0.2 a	14.6 ± 0.4 a
	2000	147.1 ± 7.4 ab	6.7 ± 0.2 b	13.8 ± 0.4 b
	3000	145.9 ± 5.0 ab	6.8 ± 0.1 b	13.8 ± 0.3 b
	4000	135.5 ± 3.6 c	6.2 ± 0.2 c	12.6 ± 0.1 c
	5000	130.7 ± 4.4 c	5.8 ± 0.2 c	12.2 ± 0.5 c

Note: Data in the table are mean ± standard, different lowercase letters indicate significant differences between different concentrations (*p* < 0.05).

## Data Availability

The data that support the findings of this study are available from the corresponding author upon reasonable request.
